# Curcumin enhances the effects of irinotecan on colorectal cancer cells through the generation of reactive oxygen species and activation of the endoplasmic reticulum stress pathway

**DOI:** 10.18632/oncotarget.16828

**Published:** 2017-04-04

**Authors:** Yan-Feng Huang, Da-Jian Zhu, Xiao-Wu Chen, Qi-Kang Chen, Zhen-Tao Luo, Chang-Chun Liu, Guo-Xin Wang, Wei-Jie Zhang, Nv-Zhu Liao

**Affiliations:** ^1^ Department of Traditional Chinese Medicine, Shunde Hospital of Southern Medical University, Guangdong 528300, China; ^2^ Department of Gastrointestinal Surgery, Shunde Women and Children's Hospital Affiliated to Jinan University, Guangdong 528300, China; ^3^ Department of Gastrointestinal Surgery, Shunde Hospital of Southern Medical University, Guangdong 528300, China

**Keywords:** curcumin, colorectal cancer, irinotecan, ROS, ER stress

## Abstract

Although initially effective against metastatic colorectal cancer (CRC), irinotecan-based chemotherapy leads to resistance and adverse toxicity. Curcumin is well known for its anti-cancer effects in many cancers, including CRC. Here, we describe reactive oxygen species (ROS) generation and endoplasmic reticulum (ER) stress as important mechanisms by which curcumin enhances irinotecan's effects on CRC cells. CRC cell lines were treated with curcumin and/or irinotecan for 24 h, and then evaluated using cell proliferation assays, cell apoptosis assays, cell cycle analysis, intracellular Ca^2+^ measurements, ROS measurements and immunoblotting for key ER stress-related proteins. We found that cell viability was inhibited and apoptosis was increased, accompanied by ROS generation and ER stress activation in CRC cells treated with curcumin alone or in combination with irinotecan. Blocking ROS production attenuated the expression of two markers of ER stress: binding of immunoglobulin protein (BIP) and CCAAT/enhancer-binding protein homologous protein (CHOP). Blocking CHOP expression using RNA interference also inhibited ROS generation. These results demonstrated that curcumin could enhance the effects of irinotecan on CRC cells by inhibiting cell viability and inducing cell cycle arrest and apoptosis, and that these effects may be mediated, in part, by ROS generation and activation of the ER stress pathway.

## INTRODUCTION

Colorectal cancer (CRC) is one of the most common cancers, and is the fourth leading cause of cancer-related death globally. The 5-year survival rate of patients with CRC metastasis is less than 10% [1]. Chemotherapy is an effective treatment for metastasized CRC, as is irinotecan, a derivative of natural camptothecin. Irinotecan alone or in combination with chemotherapy improve survival rates of patients with advanced CRC [2, 3]. Irinotecan and its active form, 7-ethyl 10-hydroxycamptothecin (SN-38), inhibit DNA topoisomerase, which induces a permanent DNA double-strand break and results in a DNA damage response (DDR). Despite the efficacy of irinotecan-based chemotherapy for CRC patients, these treatments lead to dose-dependent enterotoxigenesis and acquired chemoresistance. Therefore, novel and safe treatment strategies to overcome drug resistance and minimize side effects are needed to improve CRC treatment.

Curcumin (diferuloylmethane, C_21_H_20_O_6_) is a polyphenolic compound extracted from the plant, *Curcuma longa*. Curcumin has been extensively studied in several types of malignancies, including CRC [4–8]. Curcumin induces apoptosis, causes cell cycle arrest, and inhibits cell proliferation and invasion. In a previous study [9], we found that curcumin enhanced the efficacy of irinotecan-induced apoptosis of CRC cells (LoVo cells). We used matrix-assisted laser desorption/ionization time-of-flight mass spectrometry (MALDI-TOF) to identify related proteins and found that the combination of curcumin with irinotecan activated intracellular calcium, cellular respiration, intracellular redox reactions, and ER stress pathways *in vitro*.

Oxidative stress is important for controlling cancer cell behavior, especially cell survival and apoptosis. Oxidative stress triggers endoplasmic reticulum (ER) stress and the accumulation of misfolded or unfolded proteins in response to uncontrollable reactive oxygen species (ROS) generation, which activates ER stress-related apoptosis [10, 11]. Thus, agents that induce ROS generation and/or ER stress may be effective in promoting cell death. In this study, we determined the chemosensitization potential of curcumin in irinotecan-based chemotherapy against CRC. We demonstrated that curcumin enhances the anti-cancer effects of irinotecan *in vitro*, and curcumin alone or in combination with irinotecan inhibits cell viability and induces apoptosis through ROS generation and the activation of ER stress.

## RESULTS

### Curcumin enhanced the effects of irinotecan in inhibiting colorectal cancer cell viability

**Figure 1:**
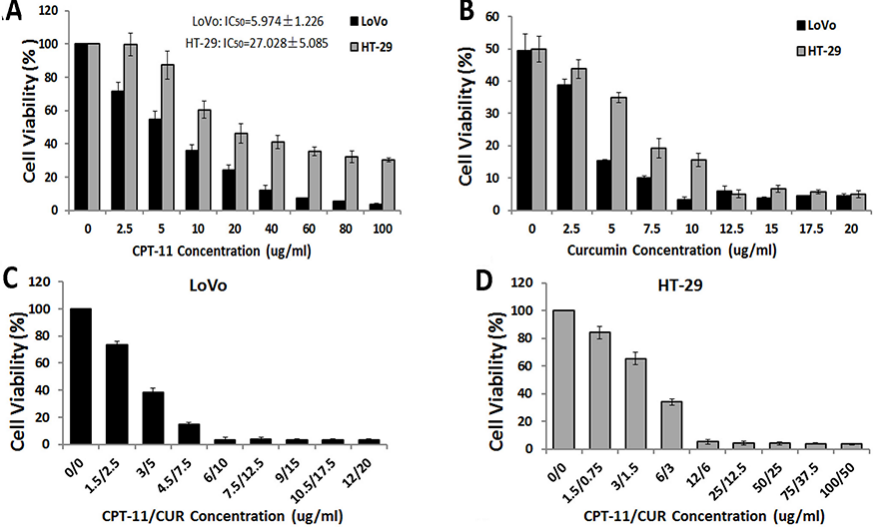
Effects of curcumin (CUR) and/or irinotecan (CPT-11) on cell viability in human CRC cell lines (**A**) The effects of irinotecan on cell viability in human CRC cells. LoVo cells or HT-29 cells were treated with irinotecan at different concentration ranges as indicated for 24 h, and then cell viability was determined by CCK-8 assay. (**B**) The effects of curcumin with irinotecan at IC_50_ concentration on cell viability in human CRC cells. LoVo cells or HT-29 cells were co-treated with irinotecan at IC_50_ concentration and curcumin at different concentration ranges as indicated for 24 h to determine the optimal ratios of the combination treatment. LoVo cells (**C**) or HT-29 (**D**) cells were treated with different concentrations of curcumin and irinotecan with the optimal ratios for 24 h to determine the optimal dose for the combination treatment (LoVo: irinotecan:6 μg/ml and curcumin: 10 μg/ml; HT-29: irinotecan: 12 μg/ml and curcumin: 6 μg/ml). Values are means ± SEM.

A dose-response study was performed with curcumin and irinotecan in LoVo and HT-29 CRC cell lines. The IC_50_ values (50% cell growth inhibitory concentrations) of irinotecan were determined (LoVo: 5.974 ± 1.226 μg/ml, HT-29: 27.028 ± 5.085 μg/ml) (Figure 1A). The cells were then exposed to their respective IC_50_ concentration of irinotecan with different concentrations of curcumin (0, 2.5, 5, 7.5, 10, 12.5, 15, 17.5 and 20 μg/ml) to determine the optimal ratio of the combination treatment by CCK-8 assay. The optimal ratios of combination treatment (irinotecan: curcumin) were 3:5 in LoVo cells and 2:1 in HT-29 cells (Figure 1B). Finally, cells were treated with different concentrations of irinotecan and curcumin at the optimal ratios, and the optimal doses for the combination treatment were determined as follows: for LoVo cells, irinotecan at 6 μg/ml and curcumin at 10 μg/ml, and for HT-29 cells, irinotecan at 12 μg/ml and curcumin at 6 μg/ml (Figure 1C and 1D). These concentrations were used in all subsequent experiments. Cell viability with combination treatment was lower than irinotecan treatment alone (LoVo: 6 μg/ml and HT-29: 12 μg/ml). These results suggested that curcumin with irinotecan caused further inhibition of growth in both LoVo and HT-29 CRC cell lines.

### Curcumin increased intracellular calcium and induced apoptosis and cell cycle arrest in colorectal cancer cells

**Figure 2:**
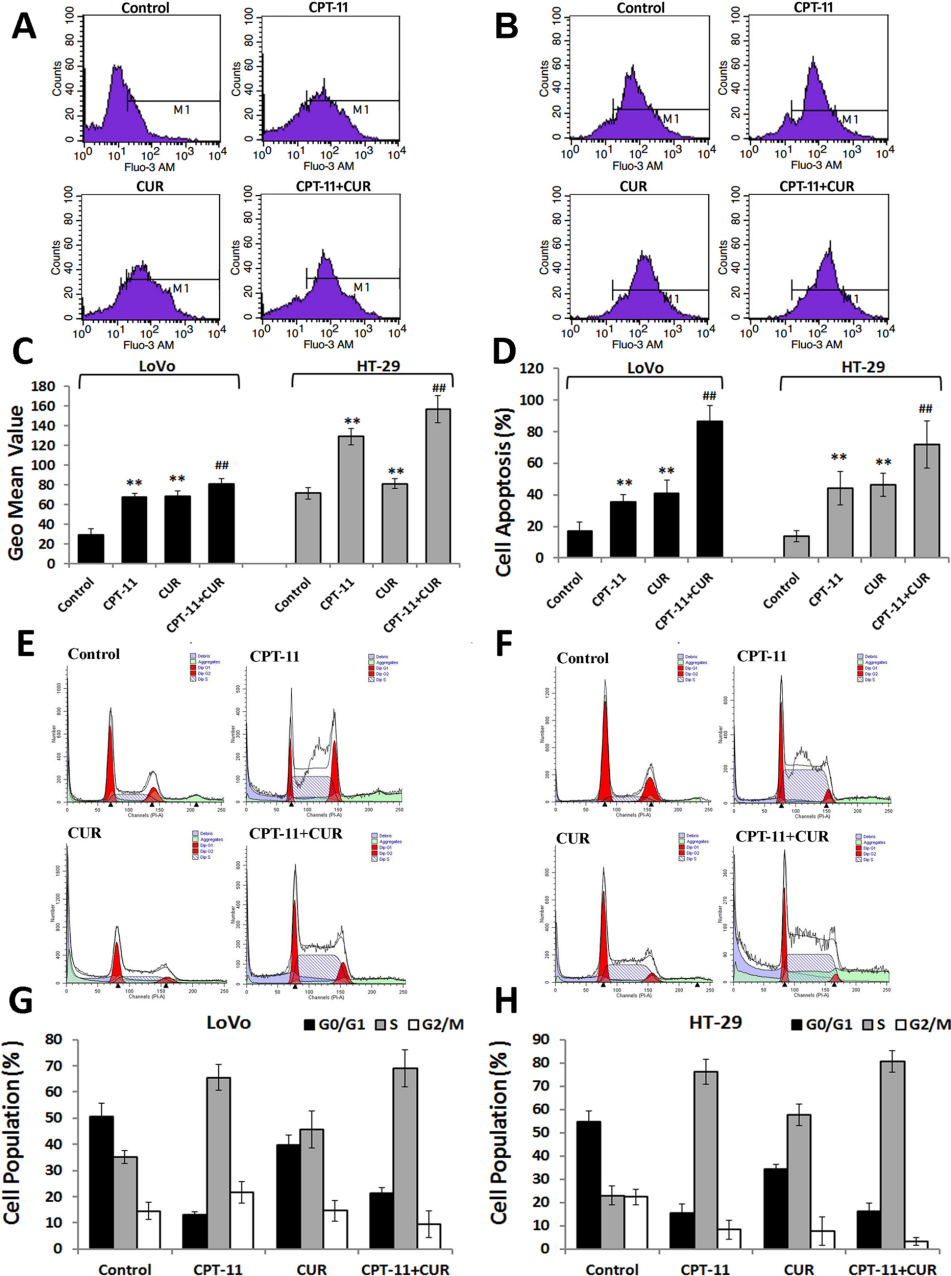
Effects of curcumin and/or irinotecan on intracellular calcium, apoptosis, and cell cycle arrest in human CRC cell lines (**A**, **B**) LoVo cells or HT-29 cells were treated with curcumin and/or irinotecan at the optimal concentrations for 24 h, and then cells were stained with Fluo-3/AM probes and analyzed by flow cytometry. (**C**) Graphs show the geometric mean values of fluorescence intensity. (**D**) LoVo cells or HT-29 cells were stained with Annexin V-FITC/PI and analyzed by flow cytometry. (**E**, **F**) The number of cells in G2/M phase was determined via flow cytometry. (**G**, **H**) Distribution of MCF 7 cells in various phases of the cell cycle. Values are means ± SEM. **< 0.01versus control group; ^##^< 0.01versus irinotecan group.

Calcium (Ca^2+^) is a second messenger involved in many cellular processes, and levels of Ca^2+^ are increased during both early and late stages of apoptosis. Both irinotecan and curcumin increased the levels of intracellular Ca^2+^ in LoVo and HT-29 cells. However, intracellular Ca^2+^ levels were increased more in cells treated with the combination of curcumin and irinotecan compared to those treated with irinotecan alone (*P* < 0.01 for both; Figure 2A–2C).

To examine the mechanisms by which curcumin and/or irinotecan inhibit the CRC cell viability, we used flow cytometry to examine their effects on apoptosis and cell cycle arrest. After treatment with curcumin, irinotecan, or the combination, apoptosis was observed in both LoVo and HT-29 cells. The combination of curcumin and irinotecan caused increased apoptosis when compared with the treatment with the individual agents (*P* < 0.01 for both; Figure 2D). There was also a reduction of cells in the G0/G1-phase in both single and combined treatment groups, and an accumulation of cells in the S-phase of the cell cycle, in both cell lines (Figure 2E–2H). Taken together, these results indicated that curcumin and/or irinotecan treatment could induce apoptosis and cell cycle arrest in CRC cells.

### Curcumin and/or irinotecan triggered reactive oxygen species generation in colorectal cancer cells

**Figure 3:**
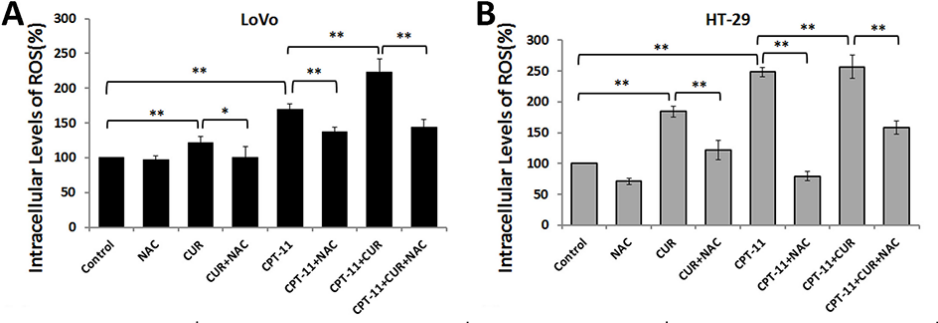
Effects of curcumin and/or irinotecan on ROS generation in human CRC cell lines After pre-treatment with 5 mM NAC for 2 h, LoVo cells (**A**) or HT-29 cells (**B**) were treated with curcumin and/or irinotecan at the optimal dose concentrations for 24 h, and cellular ROS levels were measured with 10 μM DCFH-DA using a fluorescence microplate reader. Values are means ± SEM. *< 0.05; **< 0.01.

Curcumin triggers ROS generation in several different tumor cells [10, 12, 13], which might be an effective strategy to eliminate cancer cells. We next investigated whether curcumin induced intracellular ROS generation in CRC cells. Intracellular ROS levels were assessed by dichloro-dihydro-fluorescein diacetate (DCFH-DA) assay. Curcumin and/or irinotecan treatment increased ROS generation in LoVo and HT-29 cells, compared to the control groups (*P* < 0.01 for both). However, the effect was much more pronounced in the combination treatment groups (*P* < 0.01 for both). Conversely, pretreatment with the ROS scavenger N-acetyl-L-cysteine (NAC, 5 mM) 2 h prior to treatments, inhibited ROS generation in both CRC cell lines (Figure 3). These results demonstrated that both irinotecan and curcumin treatments trigger ROS production in CRC cells, and that curcumin enhances the effects of irinotecan on ROS generation.

### Curcumin enhanced the anti-tumor activity of irinotecan through reactive oxygen species generation

**Figure 4:**
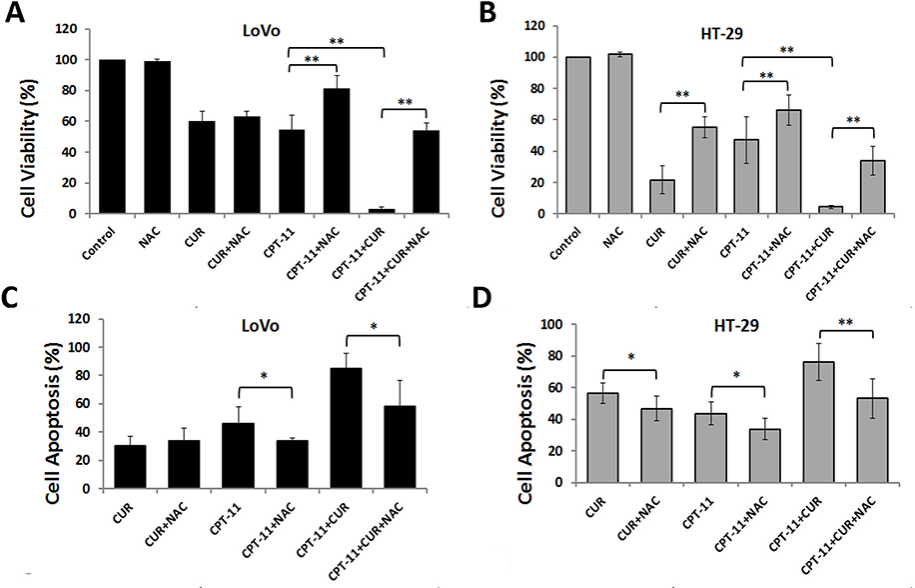
Anti-colorectal cancer effects of curcumin and/or irinotecan are dependent on ROS (**A**, **B**) The effects of NAC on cell growth inhibition induced by curcumin and/or irinotecan. After pretreatment with 5 mM NAC for 2 h, LoVo cells (A) or HT-29 cells (B) were treated with curcumin and/or irinotecan for 24 h, then cell viability was assessed by CCK-8 assay. (**C**, **D**) The effects of NAC on apoptosis induced by curcumin and/or irinotecan. After cells were treated as described above, cell apoptosis was measured by Annexin V-FITC/PI staining. Values are means ± SEM. *< 0.05; **< 0.01.

Oxidative stress plays an important role in controlling cancer cell behavior, and high levels of ROS increase cancer cell apoptosis [14, 15]. We examined whether increased ROS was required for reduced cell viability and increased apoptosis of LoVo and HT-29 cells with curcumin and/or irinotecan treatment. The growth inhibitory effects of individual and combination treatments were all blocked by NAC pretreatment (Figure 4A and 4B). As shown in Figure 4Cand 4D, NAC also blocked the cell apoptosis induced by curcumin and/or irinotecan. These results demonstrated that cell growth inhibition and apoptosis induced by combination treatment might partly depend on ROS production.

### Curcumin alone or combined with irinotecan activated endoplasmic reticulum stress in colorectal cancer cells

**Figure 5:**
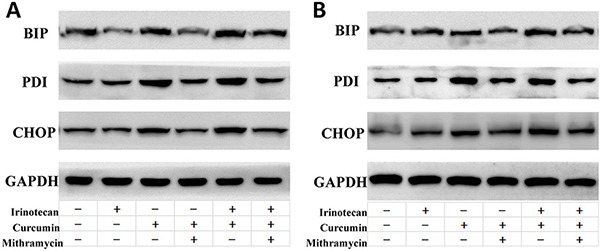
Effects of ER stress inhibitors on ER stress induced by curcumin and/or irinotecan in human CRC cell lines After pretreatment with 0.1 μM mithramycin (MTM) for 30 min, LoVo cells (**A**) or HT-29 cells (**B**) were treated with curcumin and/or irinotecan at the optimal dose concentrations for 24 h, and expression of BIP, PDI, and CHOP were assessed by western blotting.

Increased ROS generation or oxidative stress could increase protein mis-folding, subsequently activating ER stress and leading to apoptosis [10, 11]. We therefore determined the effects of treatment with curcumin and/or irinotecan on the induction of ER stress. Curcumin increased the expression of ER stress-associated proteins binding of immunoglobulin protein (BIP), protein disulfide isomerase (PDI), and CCAAT/enhancer-binding protein homologous protein (CHOP) in LoVo and HT-29 cells. Combination treatment increased the level of these proteins higher than curcumin treatment alone. There was also still a difference between irinotecan treatment and control groups. Mithramycin is a gene-selective SP1 inhibitor that blocks transcription and protein synthesis of ER chaperones [16]. Mithramycin treatment down-regulated the protein expression induced by curcumin or combination treatment in LoVo and HT-29 cells (Figure 5). Together, these results showed that curcumin alone or in combination treatment activates ER stress in CRC cells.

### Curcumin alone or combined with irinotecan reduced cell viability and induced cell apoptosis through the activation of er stress pathways

**Figure 6:**
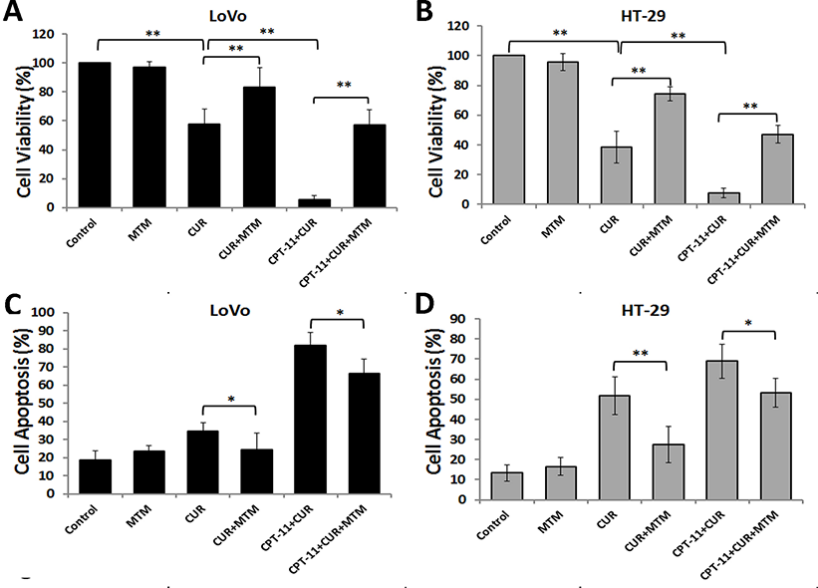
ER Stress is mediates the anti-colorectal cancer effects of curcumin alone or combined with irinotecan (**A**, **B**) The effects of an ER stress inhibitor on cell growth inhibition induced by curcumin alone or with irinotecan. After pretreatment with 0.1 μM mithramycin (MTM) for 30 min, LoVo cells (A) or HT-29 cells (B) were treated with curcumin alone or with irinotecan for 24 h, then cell viability was assessed by CCK-8 assay. (**C**, **D**) The effects of an ER stress inhibitor on apoptosis induced by curcumin alone or with irinotecan. After cells were treated as described above, cell apoptosis was measured by Annexin V-FITC/PI staining. Values are means ± SEM. *< 0.05; **< 0.01.

ER stress regulates various cellular pathological processes, including the induction of cancer cell apoptosis, making it a novel signaling target for the development of cancer drugs [17, 18]. Therefore, we examined cell viability and cell apoptosis of LoVo and HT-29 cells pretreated with mithramycin followed by curcumin and/or irinotecan treatment to confirm the role of ER stress in cell death. The ER stress inhibitor, mithramycin, attenuated cell growth inhibition (Figure 6A and 6B) and apoptosis (Figure 6C and 6D) caused by curcumin alone or combined with irinotecan in both CRC cell lines. These results indicated that ER stress may be involved in cell growth inhibition and apoptosis induced by curcumin in CRC cells.

### Curcumin alone or combined with irinotecan induced cell apoptosis through endoplasmic reticulum stress-mediated chop expression in colorectal cancer cells

**Figure 7:**
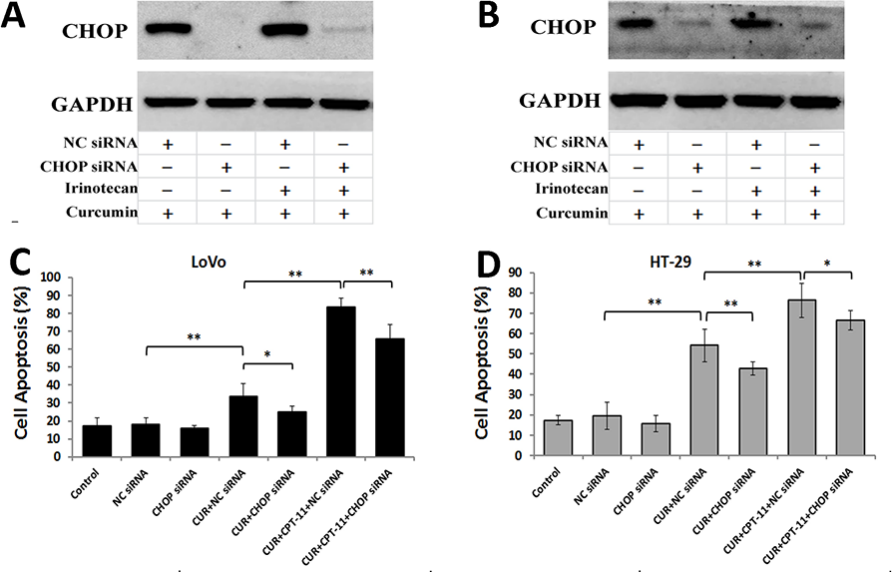
Curcumin alone or combined with irinotecan induces cell apoptosis through ER stress-mediated CHOP expression in human CRC cell lines (**A**, **B**) The inhibition efficiency of siRNAs against CHOP. LoVo cells (A) or HT-29 cells (B) were transfected with siRNAs targeting CHOP, followed by treatments with curcumin alone or with irinotecan for 24 h, and the protein levels of CHOP was assessed by western blotting. (**C**, **D**) The effects of CHOP siRNA on cell apoptosis induced by curcumin alone or with irinotecan. LoVo cells (C) or HT-29 cells (D) were treated as described above, and then cell apoptosis was measured by Annexin V-FITC/PI staining. Values are means ± SEM. *< 0.05; **< 0.01.

CHOP expression is a marker of a cell's commitment to ER stress-induced apoptosis. To confirm the role of CHOP in apoptosis induced by curcumin alone or combined with irinotecan in CRC cells, we constructed a siRNA to silence the CHOP gene. Western blots demonstrated that the transfection of the siRNA resulted in a decrease in CHOP expression in LoVo cells (Figure 7A) and HT-29 cells (Figure 7B) treated with curcumin alone or combined with irinotecan. Furthermore, when CHOP expression in CRC cells was silenced, cell apoptosis induced by curcumin, either alone or in combination with irinotecan, was reduced (Figure 7C and 7D). It is worth noting that, although the siRNA inhibited the expression of ER stress-related protein, the effect of combination treatment on cell apoptosis was not totally blocked. Taken together, these results suggested that cell apoptosis induced by curcumin alone or combined with irinotecan might involve the ER stress pathway.

### The interaction between reactive oxygen species generation and endoplasmic reticulum stress induced by curcumin alone or combined with irinotecan in colorectal cancer cells

**Figure 8:**
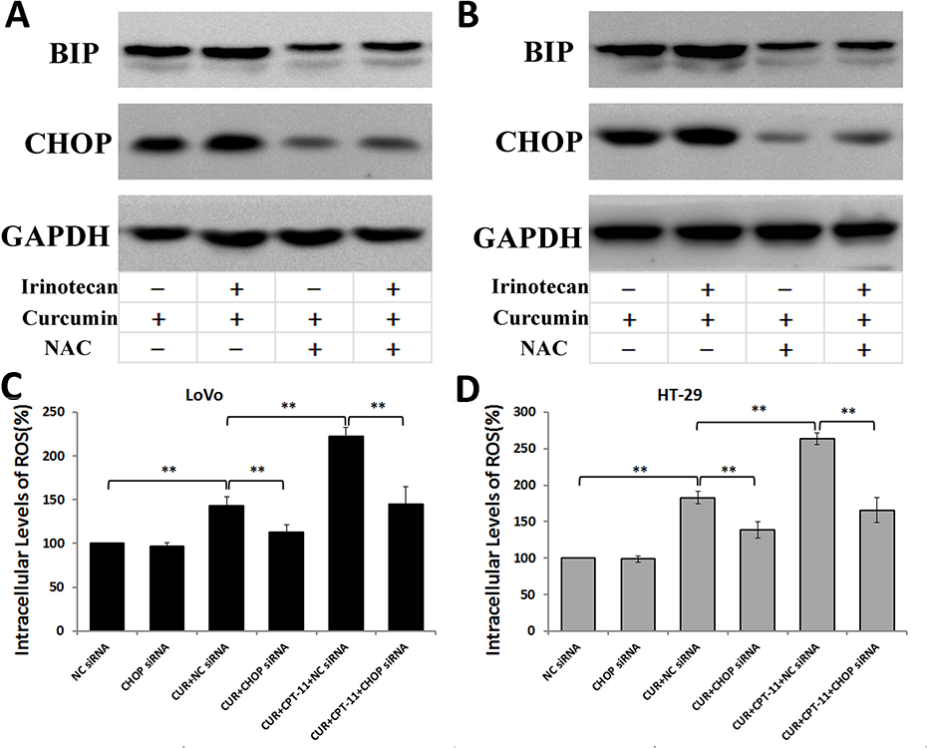
The interaction between ROS Generation and ER Stress induced by curcumin alone or combined with irinotecan in human CRC cell lines (**A**, **B**) Effects of NAC on ER stress induced by curcumin alone or combined with irinotecan. After pretreatment with 5 mM NAC for 2 h, LoVo cells (A) or HT-29 cells (B) were treated with curcumin alone or combined with irinotecan for 24 h, then BIP and CHOP expression assessed by western blotting. (**C**, **D**) Effects of CHOP siRNA on ROS generation induced by curcumin alone or combined with irinotecan. LoVo cells (C) or HT-29 cells (D) were transfected with siRNA targeting CHOP, followed by treatment with curcumin alone or with irinotecan for 24 h, and then cellular ROS levels were assessed. Values are means ± SEM. *< 0.05; **< 0.01.

Oxidative stress activates ER stress-related apoptosis [10, 11]. To determine whether ROS generation is required for ER stress induced by curcumin alone or combined with irinotecan, we first used the ROS scavenger NAC to block ROS generation, and then examined expression of ER stress protein markers. As shown in Figure 8, pretreatment with NAC inhibited the expression of ER stress markers BIP and CHOP that was induced by curcumin alone or combined with irinotecan in LoVo cells (Figure 8A) and HT-29 cells (Figure 8B). To determine whether ER stress was involved in regulating ROS generation, we silenced CHOP gene expression using siRNA and measured intracellular ROS levels in LoVo and HT-29 cells treated with curcumin alone or combined with irinotecan. Blocking CHOP expression with siRNA decreased the level of ROS production induced by curcumin alone or combined with irinotecan treatment in CRC cells (Figure 8C and 8D). These results suggested that blocking ER stress suppresses ROS production induced by curcumin alone or combined with irinotecan, and that ROS generation might be promoted by severe ER stress in CRC cells.

## DISCUSSION

In our previous studies, curcumin enhanced the efficacy of irinotecan-induced apoptosis in LoVo cells. Proteomic analysis through MALDI-TOF/TOF identified 11 repeated protein nodes, which are involved in intracellular calcium pathways, intracellular redox reaction pathways, and intracellular endoplasmic reticulum (ER) stress [9]. In this study, we demonstrated the curcumin enhanced the effect of irinotecan against CRC cells through ROS generation and activation of the ER stress pathway. First, we determined the optimal dose for the combination treatment of curcumin and irinotecan in LoVo cells and HT-29 cells, respectively. We found that the combination treatment exhibited greater anti-cancer effects, including decreased cell viability and increased cell apoptosis/cell cycle arrest, when compared with the single drug treatments. Furthermore, we showed that curcumin and/or irinotecan induced ROS-dependent apoptosis and reduced cell viability in two human CRC cell lines. Curcumin alone or with irinotecan triggered ER stress, which could be inhibited by blocking ROS generation. Furthermore, ROS production induced by curcumin alone or with irinotecan could be suppressed by blocking ER stress.

Cell cycle analysis showed that untreated LoVo and HT-29 cells underwent normal cell cycles with a transitory S phase and a high proportion of cells in G1 phase. After treatment with curcumin or/and irinotecan, cells accumulated in S phase and subsequently became apoptotic, instead of proceeding from S to G2 phase. The S phase of the cell cycle is connected with the major cellular event of replication [19]. Irinotecan and its active form SN-38 specifically inhibit DNA topoisomerase, inducing S cell cycle arrest in CRC cells [20, 21]. Curcumin has also been reported to arrest the cell cycle at S phase in CRC cells [22]. As curcumin and irinotecan both arrested the cell cycle in the S phase in CRC cells, combination treatment might be a better therapeutic choice than curcumin or irinotecan alone.

Accumulation of ROS can result in oxidative stress and play a key role in controlling cancer cell behavior. While cancer cells may potentially benefit from oxidative stress because of the increased rate of mutations [23, 24], cancer cells have a higher basal level of oxidative stress than non-malignant cells, making them vulnerable to the acute induction of oxidative stress [24, 25]. High levels of ROS can trigger a series of pro-apoptotic signaling pathways, including ER stress and mitochondrial dysfunction, which ultimately leads to impairment of cell function and necrosis or apoptosis [26]. ROS-mediated pathways play an important role in cell apoptosis induced by curcumin in various different cancer cells [10, 11, 13]. In our previous study, we found that curcumin enhanced the efficacy of irinotecan-induced apoptosis, possibly due to proteomic changes of redox reaction pathways [9]. Here, we found that curcumin and/or irinotecan increased ROS levels in LoVo and HT-29 cells, and combination treatment induced higher ROS levels than irinotecan or curcumin alone. In addition, the increased ROS was accompanied by increased apoptosis. Importantly, NAC pretreatment almost completely reversed cell growth inhibition and apoptosis, suggesting that ROS generation, at least partially, is required for cell growth inhibition and apoptosis in CRC cells.

The ER plays a critical role in the regulation of protein synthesis, folding, and trafficking. Agents or pathological conditions, such as oxidative stress, which adversely affect ER protein folding, can cause the accumulation of unfolded or misfolded proteins, and amplify unfolded protein response (UPR) signaling [27]. High levels of ROS accumulation can cause ER stress and ER stress-dependent cell apoptosis, while antioxidants reduce ER stress and improve cell survival. However, prolonged ER stress can induce the generation of ROS and unfolded protein in the ER lumen, which signals ROS production as a second messenger to activate the UPR and induce apoptosis [28, 29]. In our study, we found that curcumin alone or with irinotecan increased the expression of BIP, PDI, and CHOP, which mediate ER stress in CRC cells. Blocking CHOP with RNA interference or the ER stress inhibitor mithramycin dramatically decreased cell apoptosis, suggesting apoptosis induced by curcumin alone or with irinotecan is mediated, at least partially, by ER stress. Moreover, we found that blocking ROS production with the ROS scavenger NAC attenuated the BIP and CHOP expression induced by curcumin alone or with irinotecan. Conversely, blocking CHOP with RNA interference inhibited ROS generation, suggesting that ER stress increased ROS production, in turn. Our study demonstrated that the interaction between ROS generation and ER stress induced by curcumin alone or combined with irinotecan contributes to apoptosis in CRC cells.

In conclusion, the data presented in this study demonstrate that curcumin enhances the effect of irinotecan against CRC cells by inducing cell cycle arrest and apoptosis, and inhibiting cell viability. More importantly, these alterations were mediated by ROS generation and activation of the ER stress pathway. Overall, our results indicate that combination treatment with curcumin and irinotecan could provide an improved strategy for CRC chemotherapy.

## MATERIALS AND METHODS

### Cell culture and treatment

Human colorectal cancer cell lines, LoVo and HT-29, were obtained from the American Type Culture Collection (Manassas, VA, USA). Cells were grown in DMEM (Gibco, Brazil) supplemented with 10% fetal bovine serum (Gibco, Brazil), penicillin (100 U/mL), and streptomycin (100 μg/mL) at 37°C in 5% CO_2_. Cells in mid-logarithmic growth were used for experiments. A stock solution of curcumin was prepared in DMSO and an equal volume of DMSO (final concentration 0.1%) was added to the control. When cells reached 75% confluences, they were treated with the indicated concentration of curcumin and/or irinotecan. When indicated, N-acetyl-L-cysteine (NAC, 5 mM) and mithramycin (0.1 μM) were added 2 h and 30 min, respectively, before curcumin and/or irinotecan administration.

### Cell viability assay

The effect of irinotecan, curcumin and their combination on viability of LoVo and HT-29 cells was determined by the Cell Counting Kit-8 (CCK-8) as described previously [30]. Briefly, the cells (2,500 per well) were exposed to different concentrations of irinotecan, in a 96-well plate for 24 h at 37°C to determine the IC_50_ values (50% cell growth inhibitory concentrations). Then cells were co-treated with IC_50_ concentration of irinotecan and different concentrations of curcumin (0, 2.5, 5, 7.5, 10, 12.5, 15, 17.5 and 20 μg/ml) for 24 h to determine the optimal ratio of the combination treatment. Additionally, cells were treated with different concentrations of curcumin and irinotecan with the optimal ratios for 24 h to determine the optimal dose for the combination treatment. After treatment, 10 μl CCK-8 was added to each well, and the plate was incubated at 37°C for 2 h. Optical density (OD) values were assessed at 450 nm with the microplate reader (Fluoroskan Ascent; Thermo Fisher Scientific, Waltham, MA, USA). Cell viability was expressed as percentage of the vehicle controls. All experiments were performed in triplicate and repeated three times.

### Flow cytometric determination of Ca^2+^

The levels of free cytosolic calcium were measured using the cell-permeable calcium-sensitive fluorescent dye Fluo-3/AM (Beyotime, Shanghai, China). Cells were exposed to various treatments as indicated for 24 h. After washing with ice-cold PBS, cells were detached in trypsin and centrifuged (5 min, 4°C, 2000 rpm), followed by resuspension in PBS. Then the cells were incubated with 5 mM Fluo-3/AM for 30 min at 37°C. The fluorescence intensity of Fluo-3/AM probes was analyzed by a FACScan flow cytometer (Becton Dickinson, Franklin Lakes, NJ, USA).

### Quantification of apoptotic cells

Annexin V/P staining was used to quantify the effect of irinotecan, curcumin and their combination on apoptosis with Annexin V-FITC Apoptosis Detection kit (KeyGEN, Nanjing, China). After treatment, cells were washed with cold PBS twice, then detached, centrifuged and resuspended as described above. Cells were then centrifuged again and resuspended in 200 μl 1X Annexin binding buffer. Subsequently, cells were incubated with Annexin V-FITC (2.5 ml) and propidium iodide (5 ml) for 15 min at room temperature. Samples were analyzed for apoptosis by a FACScan flow cytometer (Becton Dickinson, Franklin Lakes, NJ, USA).

### Cell cycle analysis by flow cytometry

After the indicated treatments, cells were fixed using ice cold 70% ethanol, washed with PBS twice, and then resuspended with propidium iodide (10 mg/ml) and ribonuclease A (0.1%) in PBS for 30 min. Cells were incubated for 30 min in the dark at room temperature, then analyzed with a FACScan flow cytometer (equipped with a 488 nm argon laser) to measure the DNA content. Data were obtained and analyzed with Cell Quest Software (Becton Dickinson, Franklin Lakes, NJ, USA). Experiments and analyses were performed in triplicate.

### Reactive oxygen species (ROS) assay

The fluorescent probe 2′, 7′-dichloro dihydrofluorescein diacetate (DCFH2-DA, Sigma) was used to assess the production of ROS. Briefly, cells in 96-well plates were exposed to different treatments for 24 h. After washing with PBS twice, cells were incubated with DCFH-DA (10 mM) at 37°C for 30 min in the dark. To quantitate ROS levels, fluorescence was detected with a microplate reader (Fluoroskan Ascent; Thermo Fisher Scientific, Waltham, MA, USA) at excitation and emission wavelengths of 485 and 528 nm, respectively, in triplicate.

### Western blotting

Cells were lysed with ice-cold cell lysis buffer. Protein concentrations were determined using a BCA protein assay kit (Pierce, number 23225). Equal aliquots (30 μg) of protein samples were applied to 10% SDS-PAGE gels, transferred to polyvinylidene fluoride (PVDF) membranes, and blocked with 5% skim milk TBST (Tris-buffered Saline Tween-20) buffer. The membranes were incubated with primary antibodies including anti-BIP, anti-PDI, anti-CHOP (1:1000; Proteintech, USA); anti-GAPDH (1:1000; Santa Cruz Biotechnology) at 4°C overnight. Then the membranes were incubated with anti-rabbit or anti-mouse antibodies at room temperature for 1 h. The protein bands were captured and documented through gel image analysis system (ChemiDox XRS, Bio-Rad, USA). The intensities of the protein bands were analyzed by Molecular Imaging Software Version 4.0, which was provided by Kodak 2000 MM System. GADPH protein was used as the internal control.

### CHOP siRNA synthesis and cell transfection

To silence gene expression by RNA interference, cells were transfected with siRNAs using Lipofectamine 3000 Transfection Reagent (Invitrogen, Madison, WI, United States) according to the manufacturer's instructions. Transfected cells were grown at 37°C for 6 h, followed by incubation with complete medium. The sequences for CHOP siRNAs and control siRNA were as follows: CHOP-1: sense: 5′-CUACCAGAAAGGUAUACCUTT-3′, anti-sense:5′-AGGUAUACCUUUCUGGUAGTT-3′; and CHOP siRNA-2 sense: 5′- GACCCUUGUGCUCGUUGUCTT-3′, anti-sense: 5′-GACAACGAGCACAAGGGUCTT-3′; and the negative control siRNA: sense: 5′-UUCUCCGAACGUGUCACGUTT-3′, anti-sense: 5′-ACGUGACACGUUCGGAGAATT-3′.

### Statistical analysis

Statistical significance was estimated using one-way analysis of variance. Values with *P* < 0.05 were considered significant. Results are expressed as means ± S.E.M. and represent assays from at least three independent experiments. Statistical analyses were performed using SPSS 13.0 (SPSS, Chicago, IL, USA).
